# Neurochemical crossroads: exploring the neurotransmitter network in chronic pain and depression comorbidity

**DOI:** 10.3389/fnmol.2025.1675814

**Published:** 2025-10-03

**Authors:** Min Ma, Yue Zhang, Kunming Tao, Zhijie Lu

**Affiliations:** ^1^Department of Anesthesiology, Affiliated Hospital of Inner Mongolia Medical University, Hohhot, China; ^2^Department of Anesthesiology, Minhang Hospital, Fudan University, Shanghai, China; ^3^Renji Hospital, Shanghai Jiao Tong University School of Medicine, Shanghai, China; ^4^Key Laboratory of Anesthesiology, Shanghai Jiao Tong University, Ministry of Education, Shanghai, China; ^5^Department of Anesthesiology, Shanghai Eastern Hepatobiliary Surgery Hospital, Shanghai, China

**Keywords:** chronic pain, depression, glutamate, excitatory-inhibitory balance, GABA, endogenous opioid system, neuropeptides, neurochemical mechanisms

## Abstract

Chronic pain and depression often co-occur, exhibiting a complex, bidirectional relationship that significantly exacerbates the clinical burden and complicates treatment strategies. Recent studies have identified neurochemical mechanisms as the fundamental biological basis for this interaction. Specifically, the imbalance between excitatory glutamate and inhibitory *γ*-aminobutyric acid (GABA), dysfunction of the endogenous opioid system, and dysregulation of various neuropeptides and non-classical neurotransmitters collectively constitute the neurobiological foundation of disturbances in pain perception and emotional regulation. Glutamate-mediated synaptic excitation and the reduction of GABA’s inhibitory function contribute to central sensitization and the abnormal processing of negative emotions. The endogenous opioid system plays a critical role in alleviating pain and emotional disturbances by regulating descending pain control pathways and the limbic system, with receptor dysfunction and expression imbalance being key mechanisms in the comorbidity. Additionally, neuropeptides such as substance P, corticotropin-releasing factor (CRF), and oxytocin participate in stress responses, reward modulation, and emotional control, thereby exacerbating the pathological connection between chronic pain and depression. This review collects the most recent findings on neurochemical interactions in the comorbidity of chronic pain and depression. The goal of this summary is to further our understanding of the molecular mechanisms in this comorbidity, as well as provide theoretical support for intervening in the neurotransmitter system in a targeted way.

## 1 Introduction

Chronic pain, typically defined as pain that persists or recurs for more than 3 months, has become a significant global public health issue, imposing a considerable burden on both individuals’ quality of life and socio-economic development (Nephew et al., 2022; Yang et al., 2023). The etiology of chronic pain is complex and multifactorial, encompassing various types such as neuropathic pain, inflammatory pain, and musculoskeletal pain. Chronic pain often presents as burning sensations, electric shock-like pain, numbness, or tingling, and may be accompanied by sensory abnormalities such as tactile allodynia or hyperalgesia. Each type of pain uniquely affects the patient’s daily functioning, leading to varying degrees of functional impairment (Karimi et al., 2024; Fornasari, 2012; Zhang et al., 2022; Sha et al., 2024).

**Figure 1:**
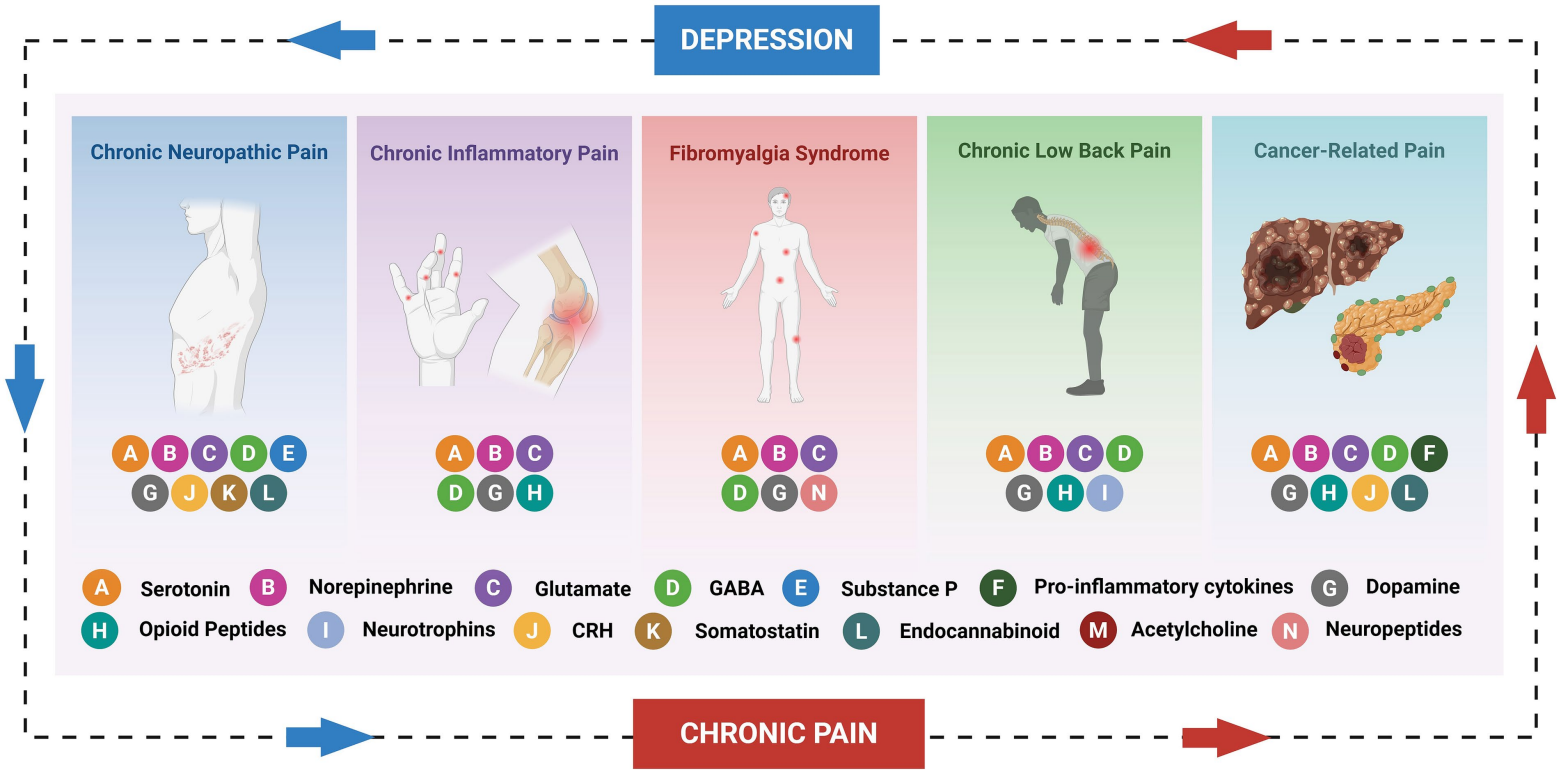
Neurochemical commonalities across chronic pain conditions contributing to depression comorbidity. Schematic illustration showing five major chronic pain conditions—chronic neuropathic pain, chronic inflammatory pain, fibromyalgia syndrome, chronic low back pain, and cancer-related pain—each associated with a distinct but overlapping neurochemical profile. Key neurotransmitters and neuromodulators are represented by color-coded symbols, including classical monoamines (e.g., serotonin (A), norepinephrine (B), dopamine (G)), excitatory and inhibitory amino acids (e.g., glutamate (C), GABA (D)), opioid peptides (H), neuropeptides (N), and others such as CRH (J), endocannabinoids (L), and pro-inflammatory cytokines (F). Arrows indicate the bidirectional interaction between chronic pain and depression, underscoring the shared neurochemical substrates underlying this comorbidity. This integrative framework highlights potential targets for cross-system therapeutic strategies.

Chronic pain and depression are highly comorbid in clinical practice, with a complex, bidirectional relationship that not only significantly impairs quality of life but also complicates treatment and increases the burden on healthcare resources (Schmaling and Nounou, 2019; Werneck and Stubbs, 2024). Epidemiological studies suggest that approximately 30 to 50% of chronic pain patients also experience depressive symptoms to varying degrees, often leading to prolonged illness, reduced treatment efficacy, and exacerbated functional impairment (Delpech et al., 2013; Aaron et al., 2025). Neuroscientific research has revealed significant overlap between chronic pain and depression, both in clinical presentation and neurobiological mechanisms, particularly regarding brain region function and neurochemical signaling. Multiple neurotransmitter systems, including monoamines (e.g., serotonin, norepinephrine, and dopamine), excitatory and inhibitory amino acid neurotransmitters (e.g., glutamate and GABA), endogenous opioid peptides, and various neuropeptides, collectively form a neurochemical network that regulates pain perception and emotional states. These neurotransmitters influence central sensitization, emotional regulation, and cognitive functions by modulating the excitatory-inhibitory balance within key brain regions such as the prefrontal cortex, anterior cingulate cortex, amygdala, hippocampus, and thalamus, thus establishing the core mechanisms of chronic pain and depression comorbidity. To provide an integrative view of the neurochemical contributors to chronic pain and its comorbidity with depression, Figure 1 summarizes the involvement of major neurotransmitters and neuromodulators across five prevalent chronic pain conditions. This schematic illustrates how shared neurochemical imbalances—including serotonergic, dopaminergic, and glutamatergic dysregulation—contribute to the bidirectional link between pain perception and emotional dysfunction, forming the neurochemical foundation for comorbidity.

This review provides a comprehensive overview of the current research on the dysfunction and interaction of these neurotransmitter systems in the comorbid state. It highlights how these systems regulate brain circuits to create the biological circuit underlying pain-emotion comorbidity. The review further investigates potential intervention methods founded on these neurochemical processes and offers theoretical support for precision treatment as well as translational research on chronic pain as well as depression comorbidity.

## 2 Serotonin system: a critical hub connecting pain perception and emotional regulation

Serotonin, also known as 5-hydroxytryptamine or 5-HT, is an important monoamine neurotransmitter in the central nervous system that is mainly synthesized from neurons of the dorsal raphe nucleus (DRN) in the brainstem. The projections of this source are vast and almost everywhere. They extend to regions such as the prefrontal cortex, amygdala, hippocampus and other limbic structures. Likewise, they also project to the dorsal horn of the spinal cord. It is important in controlling mood, pain and cognition (Wang and Nakai, 1994; Zhang H. et al., 2024). The 5-HT system is a key crossroads between emotional and nociceptive processing in the neurobiological mechanisms that underlie the comorbidity of chronic pain and depression. The 5-HT system contains a great variety of receptors with distinct functioning and complex regulatory effects. The 5-HT₁A receptor (Gi protein-coupled) is of particular importance for emotional stability and for the phenomenon of endogenous analgesia (Garcia-Garcia et al., 2014). Research indicates that the 5-HT pathway from the DRN to CeA activates the 5-HT₁A receptor, which relieves depressive-like behavior and inhibits pain sensitization, showing the potential for bidirectional regulation (Zhou et al., 2019). The governing of the Gq protein coupled 5-HT₂A receptor by a drug is complicated. In certain regions of the brain, a substantially greater activity of this receptor may trigger pain amplification while in the prefrontal cortex the activating the 5-HT₂A receptor forces the release of glutamate which enhances mood and cognitive processes with causing an antidepressant effect (Vincent et al., 2017; Luo et al., 2020). Moreover, a type of ligand-gated ion channel called the 5-HT₃ receptor is widely found in the brainstem and spinal cord, implying a role in rapid depolarization, pain response, and anxiolysis (Cortes-Altamirano et al., 2018; de Kort et al., 2022).

In the presence of chronic pain and depression, the expression and functionality of the 5-HT₁A receptor is usually compromised, whilst the 5-HT₂A and 5-HT₃ receptors experience atypical activation. This indicates abnormalities in the regulation of signalling by neurotransmitters (de Kort et al., 2022; Hao et al., 2023). The 5-HT system is also heavily involved in the descending pain modulation network comprising the DRN, RVM, and spinal cord dorsal horn, acting together with the norepinephrine (NE) system to modulate the central transmission of noxious stimuli (Tazawa et al., 2015; Moriya et al., 2019). In cases of chronic pain, lower firing rates of the DRN neurons lead to suppression of 5-HT release and damping of the descending pain pathways, which then leads to depressive-like states. Additionally, optogenetic studies have indicated that the dysfunction of the DRN → CeA → lateral habenula (LHb) circuit augments the mutual reinforcement of pain and emotion, implying that this circuit may represent a potential target for dual action (Zhou et al., 2019).

Although most peripheral 5-HT is synthesized by chromaffin cells in the gut and is unable to cross the blood–brain barrier directly, it can indirectly regulate the central 5-HT system through vagal nerve signaling and immune-mediated mechanisms, thus forming the “gut-brain-emotion” axis (Margolis et al., 2021; Hwang and Oh, 2025). This axis may play an amplifying and sustaining role in the comorbid mechanisms of chronic pain and depression, emerging as a key focus for both basic research and clinical intervention.

## 3 Norepinephrine system: a dual regulator in the arousal–analgesia pathway

Norepinephrine (NE) is a key monoamine neurotransmitter in the central nervous system, primarily synthesized and released by neurons in the locus coeruleus (LC) of the brainstem. It plays a central role in regulating arousal, emotional state, and pain perception (Hwang and Oh, 2025). The LC–NE system projects widely through axonal pathways to brain regions such as the prefrontal cortex, limbic system, thalamus, and dorsal horn of the spinal cord, creating a functional network that integrates cognition, emotion, and nociceptive processing. This system is a vital neuroregulatory structure implicated in the comorbid mechanisms of chronic pain and depression (España et al., 2024).

It has been reported that in the states of depression, NE system activity is decreased which can result in a loss of motivation, anhedonia, cognitive slowing, sleep disturbance and impaired stress response (Montgomery and Briley, 2011; Moriguchi et al., 2017). According to a recent article in frontiers, the descending pathways from the locus coeruleus to the spinal cord are inadequate in chronic pain. As a result, there is a decline in the central inhibitory control on noxious inputs, which causes a decline in the pain threshold and an increase in the pain sensitivity (España et al., 2024). Norepinephrine (NE), by activating α2-adrenergic receptors in the spinal dorsal horn, inhibits the release of excitatory glutamate, while enhancing GABAergic inhibitory transmission, producing the “suppress excitability-enhance inhibition” analgesic effect (Sanna et al., 2020).

In animal models of chronic pain, the firing frequency of LC neurons and synthesis of NE has decreased substantially, which in turn has increased anxious and depressive-like behaviors, indicating NE system dysfunction may be an important comorbidity neurobiological marker (Farahani et al., 2021). Research involving functional magnetic resonance imaging, or fMRI, have shown that the link between the LC and prefrontal cortex does not function well. This does have a close relation to a deficit in emotional regulation (España et al., 2024). In the early phases, chronic stress frequently causes overactivation of the LC system, while prolonged exposure leads to depletion of NE resources in the neurons and neuroadaptive dysfunction. This magnifies emotional disturbances and sensitivity to chronic pain, forming a typical “activation—exhaustion” pathological sequence (Lupinsky et al., 2025).

## 4 Dopamine system: imbalance in the reward pathway drives “anhedonia-driven pain”

Dopamine (DA) is a neurotransmitter that plays a critical role in the regulation of reward, motivation and cognitive functions. The ventral tegmental area (VTA) is the major site of synthesis and release of this. Dopaminergic (DA) system projects widely on mesolimbic pathway. The projections from dopaminergic (DA) system are made in important brain regions. The important brain regions include nucleus accumbens (NAc), prefrontal cortex (PFC), and amygdala. It is a neural circuit that integrates emotion, motivation and process pain (Yang et al., 2018; Cross et al., 2025).

When a person is in a state of depression, the signaling of DA in the NAc-PFC pathway gets significantly reduced. This leads to anhedonia (loss of pleasure) and less motivation. The latter two are the central neurobiological mechanisms of depression. Chronic stress reduces spontaneous firing of VTA neurons, as well as dopamine (DA) synthesis and release and D₂ receptor expression. This disorder leads to a lack of sensitivity and reactivity of the reward system (Zhang C. et al., 2024). This disruption in the reward system is linked with mood disorders. It could also be the reason behind the maintenance and aggravation of chronic pain. Imaging studies of patients suffering from chronic pain show a diminished activation in the NAc which is strongly correlated with the intensity of pain subjectively felt and negative emotional experience (Baliki et al., 2010). In addition, the DA system is essential for attentional processing and subjective evaluation of pain. When this system does not work properly, it creates a vicious cycle of “loss of motivation – increased attention on pain—increased suffering.” This aggravates the comorbidity of chronic pain and depression (Mitsi and Zachariou, 2016; Huang et al., 2022). Research findings have shown an inverse relationship between DA levels in the brain and negative emotions related to pain. Furthermore, the activity levels of the NAc and striatum were closely correlated with pain perception. This highlights the importance of the DA system in linking pain experiences to emotional responses (Scott et al., 2006; Garcia Guerra et al., 2023).

As a neurochemical intersection connecting chronic pain and depression, the DA system integrates reward processing, motivation regulation, and pain cognition. Dysfunction of the microbiome may play an important part in perpetuating the comorbidity state. Future studies could focus on the differences between DA receptor subtypes like D₁ and D₂ in their functional roles in circuit regulation, and in the plasticity of the NAc–PFC–VTA circuit. Moreover, strategies aiming at dopaminergic system DA-based such as dopamine agonists, ketamine-like rapid antidepressants and transcranial magnetic stimulation (TMS) are very promising therapeutic candidates and need further development and clinical validation.

## 5 Disruption of excitatory-inhibitory balance: bidirectional dysregulation of glutamate and GABA systems

Glutamate and gamma-Vinyl GABA are rich metabolic agents in the human body and are essential for brain growth stimulation. In order to maintain the homeostasis of the brain function, they are essential due to their role in maintenance of excitatory-inhibitory balance of the neural circuits (Obata, 2013; Lee et al., 2019). In chronic pain with depression comorbidity, plasticity of synapses is abnormal, circuits in the brain are impaired and there is an enhancement of negative emotions. The mutual worsening of the disorders is driven by this imbalance mechanism.

Through activation of NMDA receptors by glutamate, synaptic excitation occurs. Thus, glutamate is recognized to be involved in central sensitization and long-term potentiation due to its gluey nature that makes neurotransmission happen. The dorsal horn of the spinal cord and cerebral cortex are hyper-responsive to glutamate signaling in chronic pain, allowing persistent amplification and transmission of noxious stimuli (Koga et al., 2016; Chen et al., 2021). In individuals with depression, elevated levels of glutamate in the prefrontal cortex and hippocampus are associated with excitotoxicity, cognitive dysfunction, and emotional regulation abnormalities, emphasizing the importance of glutamate in the pathophysiology of mood disorders.

In contrast, GABA, the primary inhibitory neurotransmitter, helps maintain neural circuit stability by reducing neuronal firing frequency. Animal models and clinical studies have shown that in the comorbid state of chronic pain and depression, GABA levels and the expression of its synthesizing enzyme (such as glutamic acid decarboxylase 67, GAD67) are generally reduced, especially in the dorsal horn of the spinal cord, amygdala, and prefrontal cortex. This reduction impairs inhibitory regulation, exacerbating negative emotional responses and increasing pain sensitivity (Meisner et al., 2010; Vaysse et al., 2011). Within the GABA receptor family, the GABA A receptor mediates rapid postsynaptic inhibition, and its dysfunction is closely linked to anxiety, hypervigilance, and hyperalgesia. Conversely, the GABA B receptor mediates slower, regulatory inhibition, and its reduced activity is associated with depression-related symptoms such as loss of motivation, cognitive slowing, and emotional exhaustion (Möhler, 2006; Munro et al., 2011; de la Salle et al., 2024). Clinical intervention studies suggest that enhancing GABAergic function can effectively alleviate comorbid symptoms. For instance, benzodiazepines, which positively regulate GABA A receptor activity, help reduce anxiety and chronic pain, while GABA B receptor agonists like baclofen show potential dual effects in both alleviating depression and providing analgesia (Cryan et al., 2004; de Haas et al., 2008). The dynamic imbalance between glutamate and GABA systems provides a neurochemical foundation for the comorbidity of chronic pain and depression. Disruption of the excitatory-inhibitory balance not only contributes to central sensitization and neurotoxicity but also weakens the brain’s ability to regulate negative emotions and noxious stimuli. Targeted regulation of this system may become a key therapeutic strategy for managing comorbidity in the future.

## 6 Dysfunction of the opioid system: “failed reward” in comorbidity mechanisms

The endogenous opioid system is composed of various opioid peptides (e.g., *β*-endorphin, enkephalins) and their receptor subtypes—*μ* receptor (MOR), *δ* receptor (DOR), and *κ* receptor (KOR)—which are widely distributed across the spinal cord, brainstem, limbic system, and cerebral cortex. This system plays a critical role in regulating pain perception and emotional responses (Higginbotham et al., 2022). As the body’s endogenous analgesic and reward system, this network undergoes functional changes under acute and chronic pathological conditions, significantly influencing the development of comorbid chronic pain and depression.

In acute pain conditions, MOR activation effectively initiates descending pain inhibition pathways, including the periaqueductal gray (PAG)—rostral ventromedial medulla (RVM)—spinal dorsal horn pathway, thereby suppressing noxious input and reducing pain perception. However, in chronic pain patients, the expression and ligand binding capacity of MOR are significantly reduced, leading to weakened descending inhibitory function, increased pain sensitivity, and the onset of negative emotional symptoms such as depression and anxiety (Advokat, 1988). Imaging studies show that in individuals with chronic pain and depression, MOR function is downregulated in brain areas responsible for regulating mood and emotions (such as the prefrontal cortex, anterior cingulate cortex (ACC) and nucleus accumbens (NAc)). This reduction constitutes important evidence for the decreased effectiveness of opioid drugs and illustrates the neurobiological mechanisms underpinning the emotional disorders (Gómez-A et al., 2019; Wang et al., 2020).

Opioid receptor subtypes play unique roles in emotional regulation. The antidepressant effects are closely linked to the activation of DOR. Moreover, the neuroplastic changes induced by DOR improve motivation and mood states. In contrast, activation of KOR is linked to increased stress response, unpleasant emotions, and social withdrawal, making it a key contributor to anxiety and depression behaviors (Zhang et al., 2023; Zhu et al., 2023). Opioid signalling in brain areas like NAc, amygdala, and VTA is impaired in neuropathic pain conditions, which is observed from animal model studies and is associated with anhedonia, social withdrawal and depression-like behaviors. Selective activation of the MOR can reverse these symptoms to improve both the emotional and the pain state, thus the MOR is a potential target. To sum up, the body’s natural opioids have a regulatory role in pain and emotion. The malfunctioning of the brain prevents the body from producing painkillers and continues negative emotions. Utilizing the opioid system, e.g., selective activation of MOR and DOR and inhibition of KOR, may provide an exciting multi-use therapy targeting both chronic pain and depression comorbidity whilst benefiting the clinics with pain relief and uplifted mood.

## 7 Neuropeptides and non-classical neurotransmitters: emerging mechanisms in stress–reward regulation for comorbidity

Mechanisms orchestrating the interplay between chronic pain and depression are facilitated by neuropeptides and non-classical neurotransmitters. They play a key role in controlling nociceptive perception, emotional processing and management of stress, constituting an essential regulatory network which works alongside classical monoamine transmitter systems.

Substance P (SP), a major neuropeptide involved in central nociceptive transmission, modulates pain perception and emotional reactions via the neurokinin-1 receptor (NK1). In cases of ongoing pain and chronic stress, the expression of SP is considerably increased in the spinal cord dorsal horn and regions like the amygdala, and hippocampus. The upregulation enhances central sensitization and assists in the encoding and response to negative emotions causing a vicious circle where pain enhances emotional disturbance and vice-versa (Zieglgänsberger, 2019).

Corticotropin-Releasing Factor (CRF) is a key molecule responsible for modulating the hypothalamic–pituitary–adrenal (HPA) axis. This molecule remains elevated during chronic stress in the human body and causes dysregulation of the neuroendocrine system. Copper reduces the levels of excitatory AMPA receptors at the postsynaptic membrane and inhibits kinase activity while increasing phosphatase activity to reduce neuronal excitability and synaptic plasticity in the NAc. This disruption causes loss of ability to feel pleasure and apathetic behavior, thereby linking neuroendocrine to reward systems and contributing to pain–depression comorbidity (Wanat et al., 2013; Xu et al., 2024).

Oxytocin has functions of a hormone and a neurotransmitter. The prefrontal cortex, amygdala and insular cortex express oxytocin. It plays a key role in alleviating anxiety, social withdrawal, and pain sensitivity by moderating the amygdala’s excessive response to negative stimuli and enhancing emotional regulation in the prefrontal cortex. Research indicates that female individuals exhibit higher sensitivity to oxytocin-mediated regulation of pain and emotion, suggesting that oxytocin may offer gender-specific therapeutic benefits (Hurlemann and Scheele, 2016; Wahis et al., 2021).

In conclusion, neuropeptides and non-classical neurotransmitters form an independent yet highly interactive regulatory axis with classical neurotransmitter systems, amplifying, transducing, and regulating pain and emotional pathways. These systems, as key mediators linking the stress and reward systems with emotional regulation networks, provide a robust theoretical basis for the development of emerging intervention strategies targeting both chronic pain and depression comorbidity.

## 8 Discussion

**Figure 2:**
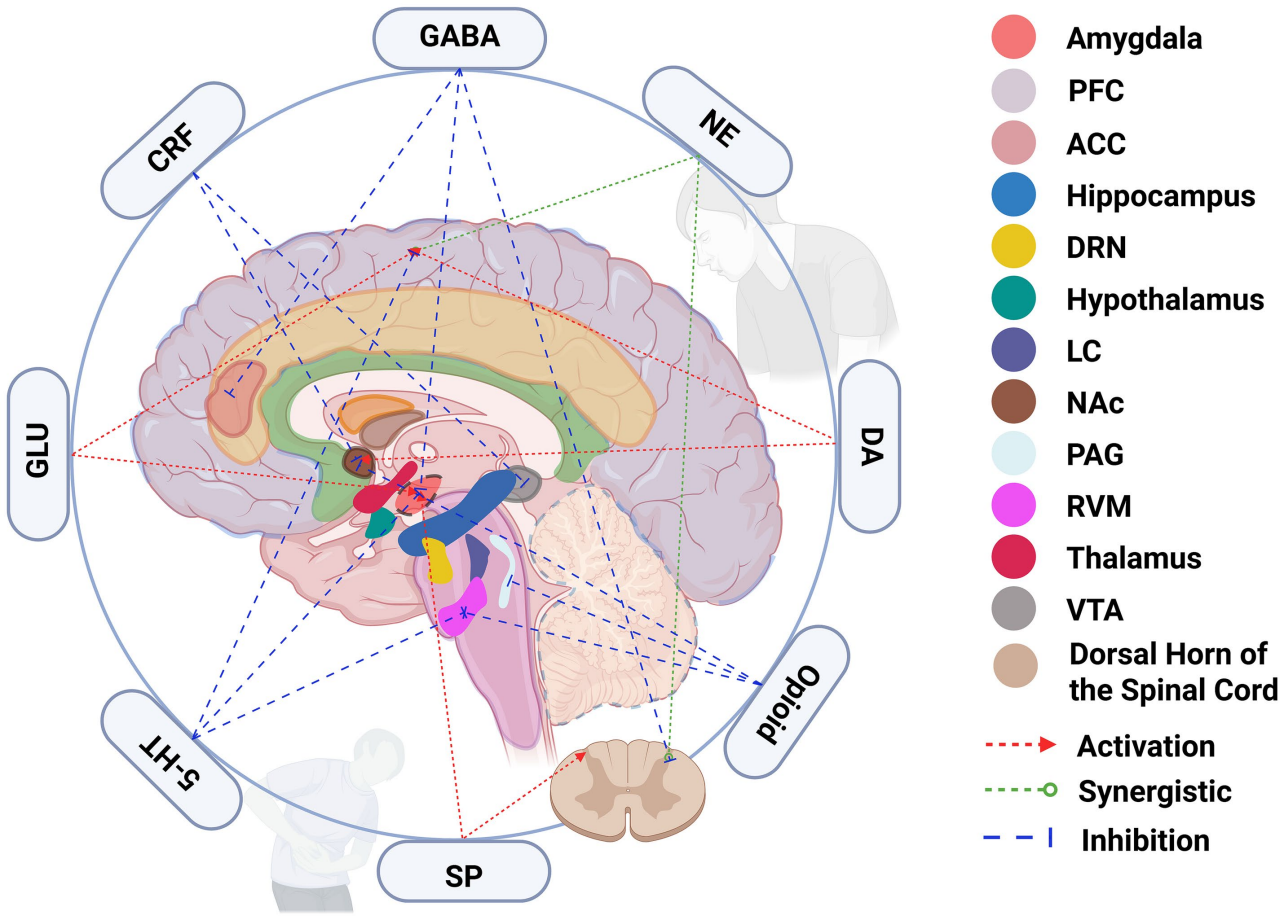
Neurotransmitter-mediated interactions among brain regions involved in chronic pain and depression comorbidity. This schematic illustrates the core neural circuitry underlying the comorbidity of chronic pain and depression, emphasizing the key roles of neurotransmitter systems in mediating inter-regional communication. Brain areas involved include the prefrontal cortex (PFC), anterior cingulate cortex (ACC), amygdala, hippocampus, dorsal raphe nucleus (DRN), locus coeruleus (LC), hypothalamus, nucleus accumbens (NAc), periaqueductal gray (PAG), rostral ventromedial medulla (RVM), thalamus, ventral tegmental area (VTA), and the dorsal horn of the spinal cord. Neurotransmitters and neuromodulators such as serotonin (5-HT), dopamine (DA), norepinephrine (NE), gamma-aminobutyric acid (GABA), glutamate (GLU), corticotropin-releasing factor (CRF), substance P (SP), and opioids regulate these circuits through excitatory (red arrows), inhibitory (blue arrows), or synergistic (green dotted lines) actions. Dysregulation of these cross-talking pathways leads to sensitization, impaired emotion regulation, and motivational deficits, establishing the neurobiological foundation of pain–depression comorbidity.

The comorbidity of chronic pain and depression represents a complex pathological condition characterized by the synergistic dysregulation of multiple neurotransmitter systems, far exceeding the dysfunction of any single neural system. Within the central descending regulatory pathways, the dorsal raphe nucleus–serotonin (DRN–5-HT) and locus coeruleus–norepinephrine (LC–NE) systems collaborate closely to regulate pain transmission and emotional responses. Dysfunction in these systems not only leads to abnormal amplification of pain processing but also disrupts the negative feedback mechanisms of emotional regulation, creating a persistent “neurofeedback failure” that exacerbates the bidirectional promotion of pain and depression (Moriya et al., 2019). The nucleus accumbens, ventral tegmental area, and anterior cingulate cortex are connected through an endogenous opioid and dopamine system that play a role in emotions and pain. The dysfunction of this network accounts for the multi-faceted clinical presentations in comorbid patients with the “anhedonia—pain sensitivity—low mood” triad that is commonly seen in so-called fibromyalgia patients. As depicted in Figure 2, chronic pain and depression comorbidity involves a dysregulated network of interacting brain regions and neurotransmitter systems. Core regions—including the PFC, ACC, amygdala, hippocampus, and brainstem nuclei—are interconnected through excitatory, inhibitory, and modulatory pathways. Key neurotransmitters such as serotonin (5-HT), dopamine (DA), norepinephrine (NE), glutamate (GLU), GABA, and endogenous opioids orchestrate cross-talk across these circuits, shaping pain perception and emotional processing. The figure illustrates how altered neurotransmitter signaling drives activation (red arrows), inhibition (blue arrows), or synergistic modulation (green dotted arrows) among nodes in the pain-emotion regulatory network.

The ACC, NAc, and PFC are key brain regions with highly integrated projections. Furthermore, the different neurotransmitter systems interacting there dynamically also have an impact on the neural circuitry process. The synaptic plastic changes that occur following chronic pain can result in a disruption of the excitatory-inhibitory balance. This can drive central sensitization of pain signals and destabilize the emotional regulation network. The ACC functions as a crucial integrative hub for emotional and pain-related information, which was associated with enhanced excitatory glutamatergic transmission and reduced GABAergic inhibition. This inequality will worsen the perception of pain and, in turn, affect emotional regulation, thus creating a negative feedback loop (Wang et al., 2020). The MOR and DOR in the nucleus accumbens mutually enhance dopamine signaling to contribute to reward and analgesic mechanisms. These imbalances in these neurotransmitter systems give rise to “reward-deficient pain” which does manifest as low mood but more importantly, the underlying neurobiological expression of the chronic pain and depression comorbidity (Zhu et al., 2023).

Importantly, the comorbidity of chronic pain and depression is highly heterogenous in nature. Certain individuals with this condition display high levels of activation of the glutamate system, resulting in central sensitization and increased pain. Others mainly have dysfunction of the serotonin (5-HT), dopamine (DA) and norepinephrine (NE) systems, with resulting major emotional disturbances (Huang et al., 2018; Trudeau and El Mestikawy, 2018; Xu et al., 2018; Li et al., 2024). The “dysfunction spectrum” emphasizes the need for precision medicine in the clinic due to the unique neurochemical and neural circuit profiles of patients, enabling stratified and personalized treatments. Gender differences are also an important variable in the management of comorbidities. The Role of Stress in Pain and Depression Pathogenesis in Women. Women are markedly more prone to pain and depression, likely due to sex-specific modulation of the oxytocin–CRF axis and a combinatorial effect of estrogens on the 5-HT system (Wahis et al., 2021). Further studies in animal models have shown that female NAc–VTA circuits are more sensitive to pain and emotional regulation, indicating that future research and therapeutic strategies should incorporate gender as a factor to improve treatment efficacy.

The comorbidity of chronic pain and depression is a widespread clinical challenge, characterized by complex interactions between the two conditions, with integrated therapeutic strategies becoming a focus of current research (Humo et al., 2019). Existing treatment approaches often prioritize alleviating individual symptoms, but there is insufficient attention given to comprehensive therapies that address the underlying neurobiological mechanisms driving both conditions concurrently (Oluboka et al., 2022; Bonilla-Jaime et al., 2022). The traditional “disease-separation—single-pathway” treatment model is insufficient for addressing the complex features of chronic pain and depression comorbidity. The emerging concept of “multi-target—systematic integration” therapy, based on the cross-regulation of neurochemical mechanisms, is gaining attention. Ketamine, as an NMDA receptor antagonist, acts on both the 5-HT and DA systems and has been proven to rapidly alleviate refractory chronic pain and depressive symptoms, demonstrating the potential of cross-neurotransmitter system interventions (Zhou and Mosadegh, 2024). Given the complexity of comorbidity, optimal treatment often requires multidisciplinary collaboration, integrating pharmacological treatment, psychological support, and social interventions (Snyder and Handrup, 2018). For patients with both pain and depression, it is crucial to assess and intervene in both conditions simultaneously to avoid the limitations of single-modality treatments and the potential risks of opioid misuse (Liao et al., 2023; Liu et al., 2024; Lottering and Lin, 2021). Furthermore, early optimization of pharmacological therapies, alongside dietary supplementation—such as oleoylethanolamide (OEA), which may restore alcohol-induced inhibition of neurogenesis and alter striatal microglial activity, thereby potentially influencing neuroinflammation and behavior—could also contribute to improved outcomes (Rivera et al., 2019).

Non-invasive neuroregulation techniques, such as transcranial direct current stimulation (tDCS), alternating current stimulation (tACS), and transcranial magnetic stimulation (TMS), target the ACC and dorsolateral prefrontal cortex (DLPFC), demonstrating dual effects of simultaneously improving both pain and emotional functions (Chen et al., 2021). Future research should focus on the regulatory mechanisms of 5-HT within the brain-gut axis, the use of CRISPR gene editing technology for precise modulation of GABA and opioid receptor subtypes, and the development of innovative therapies combining the actions of the dopamine system and oxytocin. These cross-system, multi-target therapeutic strategies hold significant promise for the systemic reorganization of neural networks, overcoming traditional treatment bottlenecks, and promoting the shift of chronic pain and depression comorbidity toward individualized precision medicine.
